# Oral Chinese patent medicines for herpangina in children: A network meta-analysis

**DOI:** 10.1097/MD.0000000000046672

**Published:** 2025-12-26

**Authors:** Yongjing Wang, Bokun Chen, Menghao Li, Yunfei Tian, Xinhui Zhang, Mingxuan He, Yidan Huo, Xiuju Liu

**Affiliations:** a Pharmacy Department, The Second Hospital of Hebei Medical University, Shijiazhuang, China; b Faculty of Social Sciences,The University of Hong Kong, Hong Kong, China; c School of Pharmacy, Hebei Medical University, Shijiazhuang, China.

**Keywords:** children, efficacy, herpangina, network meta-analysis, oral Chinese patent medicine, safety

## Abstract

**Introduction:**

Herpangina, primarily caused by Enterovirus A71 and Coxsackievirus A, is characterized by fever, oropharyngeal pain, and ulcers. The prognosis is generally favorable. However, some severe cases may lead to complications such as febrile seizures and myocarditis. In recent years, clinical studies have demonstrated that combining oral Chinese patent medicines with conventional Western medicine can improve treatment efficacy.

**Objective:**

To compare the efficacy and safety of oral Chinese patent medicines combined with conventional treatment for pediatric herpangina using network meta-analysis.

**Method:**

We systematically searched major Chinese and international databases from inception to January 1, 2024. Two researchers independently screened studies, extracted data, and assessed risk of bias (Cochrane tool). Statistical analysis was performed using Stata 16.0 and Review Manager 5.3 software. A network meta-analysis was conducted using a random-effects model. The odds ratio, and its 95% confidence interval (CI), were used as the effect measure for binary outcomes. The mean difference (MD), and its 95% CI, were used as the effect measure for continuous variables. Differences in efficacy and safety among interventions were compared and ranked independently.

**Result:**

11 oral Chinese patent medicines, 61 studies, and 6805 patients were included. The antipyretic time was ranked from best to worst as XiaoerShuangjinqingre Oral Liquid (SJQR), Lianhuaqingwen Granules (LHQW), Pudilan Anti-inflammatory Oral Liquid (PDL), XiaoerNiuhuangqingxin Powder (NHQX), Lanqin Oral Liquid (LQ), XiaoerQingyan Granules (QY), Shuanghuanglian Oral Liquid (SHL), XiaoerChaiguituire Granule (CGTR), XiaoerChiqiaoqingre Granules (CQQR), Kouyanqing Granules (KYQ), and Shufengjiedu Capsules (SFJD). The herpes regression time was ranked from best to worst as SJQR, KYQ, PDL, CQQR, SHL, NHQX, LHQW, LQ, QY, SFJD, and CGTR. The incidence of adverse reactions was ranked from lowest to highest as CQQR, PDL, LQ, CGTR, LHQW, NHQX, SJQR.

**Conclusion:**

Combining oral Chinese patent medicines with conventional treatment effectively shortens the time of fever and herpes in children. Additionally, no increase in adverse events was observed in terms of safety when oral Chinese patent medicines were combined with conventional treatment. This study has been registered in the International Prospective Register of Systematic Reviews (PROSPERO), with a registration number CRD42024503831.

## 1. Introduction

Herpangina is a specific type of acute upper respiratory infection, the primary infecting pathogens of which are Enterovirus A71 and Coxsackievirus A. The disease has a high incidence and is prevalent in the spring and summer.^[1]^ Herpes in the pharynx, oropharyngeal pain, and fever are common symptoms of this disease. The prognosis is generally favorable, but some severe cases may lead to complications such as febrile seizures and myocarditis.^[2]^ In addition, infection with Enterovirus A71, a highly neurotropic virus, can lead to life-threatening conditions such as neurogenic pulmonary edema.^[3]^ There is no specific medication available for herpangina. The standard treatment involves relieving symptoms such as fever, cough, and phlegm and correcting water, electrolyte, and acid-base imbalances. Interferon, ribavirin, and vidarabine are antiviral drugs used for causative treatment. Antibacterial medications are used for comorbid bacterial infections.^[4]^ According to traditional Chinese medicine, the disease is caused by a combination of internal and external factors, and treatment is divided into internal and external methods. External methods include acupoint application, Chinese medicine enema, acupuncture, and manipulation. Internal methods involve taking Chinese patent medicines or oral heat-clearing and detoxification formulas.^[2]^ Among them, oral Chinese patent medicines has the advantages of storage, portability, and convenience in taking medicine. In recent years, clinical studies have demonstrated that combining oral Chinese patent medicines for heat-clearing and detoxification with conventional Western medicine can improve treatment efficacy. It can be challenging to choose the correct medicine for children with different symptoms because a wide variety of oral Chinese patent medicines are available. The ingredients can be complex, and the focus of symptomatic treatment can differ between medicines. Unfortunately, no explicit clinical references or direct comparative studies guide the selection process. This study uses network meta-analysis to evaluate and rank the efficacy and safety of commonly used oral Chinese patent medicines for herpangina treatment to provide supporting evidence for clinical selection.

## 2. Resource and methods

This study has been registered in the International Prospective Register of Systematic Reviews (PROSPERO) (website: https://www.crd.york.ac.uk/prospero/ registration number: CRD42024503831). The study was written according to the Priority reporting entry for systematic evaluation and meta-analysis (PRISMA). The software used in this study includes EndNote X9 (literature management and writing) (Thomson ResearchSoft, Carlsbad), Excel 2019 (data extraction and reorganization) (Microsoft Office, Microsoft Corporation, Redmond) Review Manager 5.3 (methodology quality assessment) (The Cochrane Collaboration, Copenhagen, Denmark ), and Stata 16.0 (NMA, heterogeneity assessment, and the surface under the cumulative ranking curve [SUCRA] plot) (Stata Corporation, StataCorp LLC, Texas).

### 2.1. Inclusion criteria

#### 2.1.1. Study type and study objective

The study design is a randomized controlled trial (RCT) conducted in Chinese or English. The population to be included in the study must meet the diagnostic criteria for herpangina and must be aged between 0 and 18 years old.^[5–7]^ Patients in different intervention groups of the same study must have no significant baseline imbalance in gender, age, disease severity, and illness duration. Additionally, the 2 groups must be comparable to ensure the study’s fairness and accuracy.

#### 2.1.2. Intervention and control measure

The control group received conventional Western medicine, including anti-infection, antiviral, antipyretic, and rehydration. In contrast, the test group added an oral Chinese patent medicine based on the control group. This oral Chinese patent medicine should be 1 of the eleven kinds: Pudilan Anti-inflammatory Oral Liquid (PDL), Lanqin Oral Liquid (LQ), Shuanghuanglian Oral Liquid (SHL), XiaoerShuangjinqingre Oral Liquid (SJQR), Lianhuaqingwen Granules (LHQW), Xiaoer Chiqiaoqingre Granules (CQQR), XiaoerChaiguituire Granules (CGTR), XiaoerNiuhuangqingxin Powder (NHQX), Shufengjiedu Capsules (SFJD), XiaoerQingyan Granules (QY), and Kouyanqing Granules (KYQ).

#### 2.1.3. Outcome indicators

Time for fever reduction; time for the disappearance of herpes; incidence of adverse events (AE).

### 2.2. Exclusion criteria

Literature with incomplete data or obvious statistical errors; lack of rigour in study design; repeatedly published studies.

### 2.3. Literature search

Databases, such as China National Knowledge Infrastructure (CNKI), China Science and Technology Journal Database (VIP), Wan Fang data, PubMed, Embase, Cochrane Library, and Web of Science were searched. The Chinese database takes China National Knowledge Infrastructure as an example. The retrieval type is: SU=(“Pudilan”+“Pudilan”+“Xiaoerguqiaoqingre”+“Lanqin”+“Shuanghuanglian”+“Lianhuaqingwen”+“Lianhuaqingwen”+“Shufengjiedu”+“Chaiguituire”+“Niuhuangqingxin”+“Shuangjinqingre”+“Xiaoerqingyankeli”+“Kouyanqing”+“Koufuzhongchengyao”)*(“Paozhenxingyanyan”). The English database takes PubMed as an example, and the retrieval type is: ((Herpangina) OR (Herpetic pharyngitis)) AND ((Pudilan) OR (Chiqiaoqingre) OR (Lanqin) OR (Shuanghuanglian) OR (Lianhuaqingwen) OR (Shufengjiedu) OR (Chaiguituire) OR (Niuhuangqingxin) OR (Shuangjinqingre) OR (Qingyan) OR (Kouyanqing)). The search period is from the establishment of the database to January 1, 2024.

### 2.4. Literature screening and data extraction

Two researchers conducted the study individually according to the Inclusion and Exclusion criteria and compared the results. If there were any disputes, the decision would be referred to a third-party investigator for joint discussion. Basic information about the literature was extracted, including sample size, dosing regimen, duration of treatment, and outcome indicators.

### 2.5. Evaluation of the quality of literature

According to the Cochrane 5.0.1 systematic appraisal manual, 7 main aspects were assessed: random sequence generation, allocation concealment, implementation blinded experiment, outcome blinded experiment, completeness of outcome data, selective reporting, and other biases. “low risk” indicates a low risk of bias; “high risk” indicates a high risk of bias; “unclear risk” indicates insufficient or “unclear risk” indicates inadequate or uncertain information.

### 2.6. Statistical analysis

Stata 16.0 software was used to select a frequency-based framework random-effects model for the network meta-analysis, with the odds ratio (OR) for dichotomous variables and the mean difference (MD) for continuous variables as effect indicators. Each effect size was expressed as its 95% confidence interval (CI). Comparison-corrected funnel plots were used for publication bias testing. For each outcome indicator, the efficacy of the intervention was ranked using the area under the cumulative ranking curve (SUCRA) value. When closed loops were present in the network evidence plots, node-splitting methods were used to calculate the difference between direct and indirect comparative evidence.

## 3. Results

### 3.1. Literature search results

**Figure 1.**
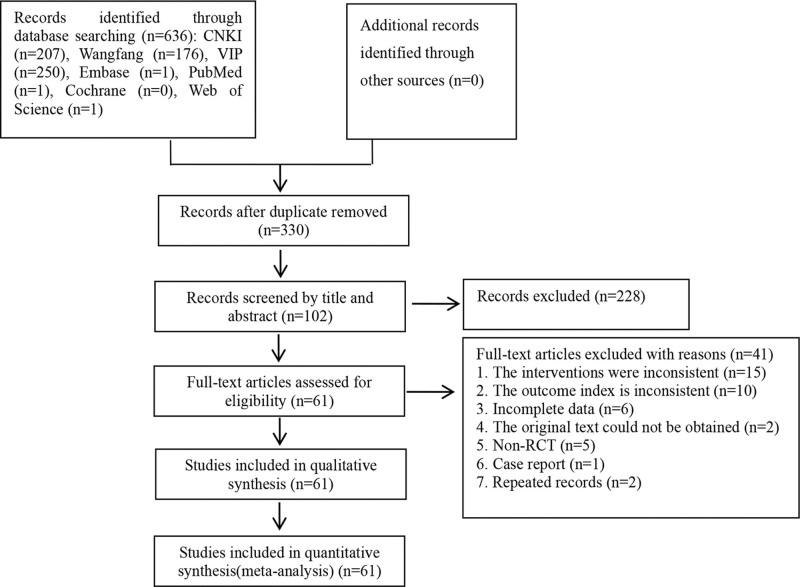
Literature screening process.

According to the inclusion and exclusion criteria, 61 RCTs were finally included.^[8–68]^ The literature screening process and results are shown in Figure 1.

### 3.2. Basic characteristics of the literature

61 RCTs, 6805 cases of sample size, 3428 cases in the test group, and 3377 cases in the control group were included. Among them, 53 reported the time to fever reduction,^[8,9,11–17,19,23–25,27–61,64–68]^ 54 reported the time to resolution of herpes,^[8,9,11–17,19,20,24,25,27–61,63–68]^ and 29 reported the incidence of AE.^[9,10,16,18–27,30,32–34,40–46,51,57–59,62]^ The basic information of the included studies is shown in Table 1.

**Table 1:** Basic information included in this study.

Publication	n/(male/female)		Age (yr) (Mean ± SD)		Course of disease (d) (Mean ± SD)		Intervention		Dose and frequency of the oral Chinese patent medicines	Treatment duration (d)	Outcomes
	T	C	T	C			T	C			
Zhu XH 2010^[8]^	50	50	0.54–6	0.54–6	–	–	PDL + A	A	5–10 mL, tid	–	*,†
Wang XL 2020^[9]^	40 (23/17)	40 (22/18)	3.5 ± 0.7	3.3 ± 0.8	1.13 ± 0.30	1.12 ± 0.27	PDL + A	A	5–10 mL, tid	5	*,†,‡
Jing FL 2015^[10]^	60 (34/26)	60 (35/25)	5.3 ± 1.2	5.3 ± 1.3	3.2 ± 1.2	3.1 ± 1.1	PDL + A	A	5–10 mL, tid	5	‡
Gan MX 2014^[11]^	48	48	0.75–7	0.75–7	<3d	<3d	PDL + A	A	5–10 mL, tid	3–5	*,†
Li ZB 2013^[12]^	100 (54/46)	82 (42/40)	0.6~2.9	0.7~2.9	–	–	PDL + A	A	3.3–10 mL, tid	5–7	*,†
Yu Y 2018^[13]^	45	43	2.6 ± 1.1	2.6 ± 1.1	0.125–3	0.125–3	PDL + A	A	3–5 mL, tid	–	*,†
Yang WQ 2017^[14]^	20 (12/8)	20 (13/7)	3.34 ± 1.45	3.36 ± 1.42	2.28 ± 0.24	2.29 ± 0.25	PDL + A	A	5–10 mL, tid	5–7	*,†
Zhang HQ 2013^[15]^	100 (59/41)	80 (43/37)	4.1	4.2	1–3	1–3	PDL + A	A	5–10 mL, tid	5	*,†
Liang ZF 2023^[16]^	100 (58/42)	100 (61/39)	3.78 ± 0.68	4.06 ± 0.52	1.03 ± 0.15	1.05 ± 0.14	PDL + A	A	3.3–10 mL, tid	5	*,†,‡
Zhang XY 2018^[17]^	36 (22/14)	36 (20/16)	2.68 ± 0.34	2.70 ± 0.32	1.56 ± 0.19	1.64 ± 0.20	CQQR + A	A	1–6 g, tid	7	*,†
Wu FC 2021^[18]^	41 (22/19)	41 (23/18)	3.21 ± 1.25	3.29 ± 1.32	1.89 ± 0.22	1.92 ± 0.25	CQQR + A	A	1–5 g, tid	3	‡
Zhang H 2015^[19]^	78 (42/36)	78 (39/39)	2.01 ± 1.26	2.21 ± 1.09	–	–	CQQR + A	A	1–4 g, tid	4	*,†,‡
Yue SW 2017^[20]^	99 (52/47)	99 (50/49)	2.9 ± 0.12	2.8 ± 0.11	–	–	CQQR + A	A	1–4 g, tid	7	†,‡
Zhu FM 2016^[21]^	63 (23/40)	63 (25/38)	2.2 ± 1.01	2.2 ± 1.3	–	–	CQQR + A	A	1–4 g, tid	–	‡
Zhang XL 2017^[22]^	60 (31/29)	60 (32/28)	0.58~6	0.58~6	–	–	CQQR + A	A	1–4 g, tid	4	‡
Li XD 2019^[23]^	50 (29/21)	50 (30/20)	3.1 ± 0.8	2.9 ± 0.9	8.4 ± 0.7	8.4 ± 2.1	CQQR + A	A	1–4 g, tid	7	*,‡
Shen TY 2020^[24]^	40 (21/19)	39 (21/18)	3.2 ± 1.4	3.4 ± 1.3	–	–	CQQR + A	A	1–4 g, tid	7	*,†,‡
Xiu WS 2021^[25]^	50 (26/24)	50 (25/25)	2.13 ± 1.45	2.67 ± 1.12	–	–	CQQR + A	A	2–4 g, tid	4	*,†,‡
Fang Y 2012^[26]^	60 (35/25)	60 (38/22)	0.5~5	0.5~5	–	–	CQQR + A	A	1–4 g, tid	–	‡
Lou SL 2019^[27]^	20 (11/9)	20 (13/7)	2.1 ± 0.2	2.2 ± 0.2	2.1 ± 0.1	2.1 ± 0.2	CQQR + A	A	2–3 g, tid	5	*,†,‡
Wang YX 2012^[28]^	40 (24/16)	40 (21/19)	0.5~6	0.5~6	<2	<2	CQQR + A	A	1–4 g, tid	4	*,†
Liu J2019^[29]^	40 (22/18)	40 (23/17)	3.3 ± 1.4	3.3 ± 1.2	<5	<5	CQQR + A	A	2–4 g, tid	5	*,†
He XY 2018^[30]^	53 (27/26)	53 (28/25)	2.15 ± 1.44	2.56 ± 1.34	–	–	CQQR + A	A	1.5–3 g, tid	4	*,†,‡
Tan RG 2018^[31]^	60 (29/31)	60 (28/32)	3.46 ± 0.42	3.51 ± 0.41	1.8 ± 0.5	1.7 ± 0.4	CQQR + A	A	2–4 g, tid	5–7	*,†
Huang ZH2022^[32]^	58 (30/28)	58 (31/27)	5.02 ± 0.66	5.01 ± 0.68	3.54 ± 0.75	3.55 ± 0.71	CQQR + A	A	2–5 g, tid	7	*,†,‡
Zhang C2023^[33]^	44 (23/21)	44 (25/19)	3.12 ± 1.25	3.10 ± 1.24	2.14 ± 0.38	2.15 ± 0.37	CQQR + A	A	1–4 g, tid	7	*,†,‡
Huang YY 2022^[34]^	40 (23/17)	40 (24/16)	3.20 ± 0.57	3.00 ± 0.55	4.56 ± 0.30	4.41 ± 0.25	CQQR + A	A	2–5 g, tid	–	*,†,‡
Shen JY 2017^[35]^	50 (27/23)	50 (25/25)	3.25 ± 1.72	3.21 ± 1.76	<2	<2	LQ + A	A	2.5–5 mL, tid	5	*,†
Su DB 2018^[36]^	60 (37/23)	60 (35/25)	4.53 ± 2.18	4.79 ± 2.05	–	–	LQ + A	A	3–15 mL, tid	14	*,†
Hu ZQ 2011^[37]^	30 (16/14)	30 (15/15)	3.2 ± 1.7	3.0 ± 1.6	–	–	LQ + A	A	5–10 mL, tid	5	*,†
Lin JY 2020^[38]^	32 (16/16)	32 (18/14)	2.03 ± 1.28	2.64 ± 1.75	1.66 ± 0.91	1.59 ± 0.87	LQ + A	A	5–10 mL, tid	5–7	*,†
Chen L 2021^[39]^	90 (51/39)	90 (48/42)	5.62 ± 0.84	5.74 ± 0.89	1.46 ± 0.34	1.47 ± 0.33	LQ + A	A	5 mL, tid	7	*,†
Liu AL 2011^[40]^	60 (33/27)	60 (32/28)	2.6	2.5	–	–	LQ + A	A	10–20 mL, tid	7	*,†,‡
Wu WY 2018^[41]^	140 (78/62)	140 (79/61)	5.01 ± 1.69	4.21 ± 1.21	–	–	LQ + A	A	6.6–10 mL, tid	5	*,†,‡
Xu CJ 2015^[42]^	45 (26/19)	45 (27/18)	1.5 ± 0.6	1.6 ± 0.4	1–3	1–3	LQ + A	A	10 mL, tid	–	*,†,‡
Huang LJ 2023^[43]^	39 (22/17)	39 (19/20)	3.16 ± 0.49	3.13 ± 0.57	1.03 ± 0.12	1.01 ± 0.14	LQ + A	A	10–20 mL, tid	5	*,†,‡
Wang HH 2023^[44]^	41 (20/21)	41 (19/22)	6.89 ± 1.83	6.23 ± 1.71	0.33 ± 0.084	0.30 ± 0.087	LQ + A	A	5 mL, tid	7	*,†,‡
Liu X 2023^[45]^	150 (90/60)	150 (85/65)	3.54 ± 1.06	3.48 ± 1.02	1.24 ± 0.45	1.23 ± 0.51	LQ + A	A	5–10 mL, tid	5	*,†,‡
Zhao TT 2023^[46]^	40 (14/26)	40 (13/27)	3.74 ± 1.05	3.70 ± 1.16	1.73 ± 0.22	1.78 ± 0.19	LQ + A	A	5 mL, tid	7	*,†,‡
Ma SL 2012^[47]^	82 (40/42)	81 (39/42)	0.5–7	0.5–7	1–3	1–3	SHL + A	A	5–10 mL, tid	3–4	*,†
Xu J 2014^[48]^	59 (35/24)	55 (33/22)	4.62 ± 1.34	4.52 ± 1.44	2.75 ± 1.46	2.91 ± 1.53	SHL + A	A	3.3–10 mL, tid	–	*,†
Yuan YY 2012^[49]^	43	43	4.5	4.5	1–3	1–3	SHL + A	A	5–10 mL, tid	3–4	*,†
Ma SZ 2015^[50]^	45 (20/25)	45 (23/22)	2.5 ± 1.2	2.5 ± 1.6	1.2 ± 0.8	1.1 ± 0.9	LHQW + A	A	1.5–3 g, tid	7	*,†
Zhang LY 2020^[51]^	47 (24/23)	43 (21/22)	2.9 ± 0.4	2.8 ± 0.3	1.2 ± 0.4	1.1 ± 0.3	LHQW + A	A	1.5–3 g, tid	7	*,†,‡
Liu XJ 2021^[52]^	38 (19/19)	38 (18/20)	2.52 ± 0.47	2.49 ± 0.51	2.13 ± 1.09	2.02 ± 1.10	LHQW + A	A	2–6 g, tid	6	*,†
Wang YL 2017^[53]^	54 (34/20)	54 (35/19)	2.3 ± 0.5	2.5 ± 0.6	–	–	LHQW + A	A	2 g, tid	7	*,†
Liu CX 2015^[54]^	37	33	2.4 ± 1.7	2.5 ± 1.4	1.3 ± 0.9	1.4 ± 0.7	SFJD + A	A	0.26–1.04 g, tid	5	*,†
Yang ML 2016^[55]^	123 (61/62)	123 (59/64)	4.2 ± 1.3	4.1 ± 1.3	1.9 ± 0.7	1.7 ± 0.8	SFJD + A	A	1.04–1.56 g, tid	5	*,†
Yang Y 2019^[56]^	35 (17/18)	35 (15/20)	4.55 ± 2.13	4.20 ± 2.47	3.44 ± 2.08	3.85 ± 2.90	SFJD + A	A	0.52–1.56 g, tid	5	*,†
Zheng GF 2016^[57]^	50 (27/23)	50 (24/26)	3.51 ± 0.90	3.33 ± 1.05	1.42 ± 0.51	1.34 ± 0.62	CGTR + A	A	4–6 g, qid	6	*,†,‡
Wu J 2019^[58]^	25 (13/12)	25 (11/14)	3.62 ± 1.55	3.33 ± 1.02	3.2 ± 1.1	32.8 ± 0.9	CGTR + A	A	5–7.5 g, qid	6	*,†,‡
Li YH 2013^[59]^	35 (19/16)	34 (18/16)	0.33~5	0.33~5	–	–	NHQX + A	A	0.3–0.6 g, bid/tid	3–5	*,†,‡
Han F 2015^[60]^	45 (24/21)	50 (26/24)	2.7 ± 1.7	2.6 ± 1.4	–	–	SJQR + A	A	5–20 mL, tid	5–7	*,†
Xu ZC 2014^[61]^	40 (22/18)	40 (24/16)	3.4 ± 1.9	3.1 ± 1.5	–	–	SJQR + A	A	10 mL, tid	–	*,†
Dai YH 2014^[62]^	80 (35/45)	80 (37/43)	0.5~12	0.5–12	–	–	SJQR + A	A	5–20 mL, tid	–	‡
Chen YC 2019^[63]^	34 (20/14)	33 (20/13)	3.11 ± 0.50	3.16 ± 0.48	4.10 ± 1.05	4.12 ± 1.02	QY + A	A	3–6 g, bid/tid	10	†
Yu DZ 2006^[64]^	48 (26/22)	48 (27/21)	0.5~7	0.5~7	1–3	1–3	QY + A	A	3–6 g, bid/tid	5	*,†
Hui XZ 2022^[65]^	34 (21/13)	34 (18/16)	4.41 ± 0.68	4.57 ± 0.77	4.76 ± 1.05	4.60 ± 1.13	QY + A	A	3–6 g, bid/tid	10	*,†
Li X2023^[66]^	44 (25/19)	44 (24/20)	7.85 ± 2.34	7.72 ± 2.45	4.16 ± 0.92	4.22 ± 0.95	QY + A	A	3–6 g, bid/tid	7	*,†
Gao SY 2020^[67]^	50 (27/23)	50 (26/24)	2.69 ± 0.74	2.69 ± 0.74	1.87 ± 0.59	1.89 ± 0.62	KYQ + A	A	1–3 g, qd	7	*,†
Liu ZY 2019^[68]^	108 (56/52)	108 (58/50)	2.65 ± 0.68	2.72 ± 0.75	1.93 ± 0.25	1.98 ± 0.28	KYQ + A	A	1–4 g, bid	7	*,†

– = no report, A = conventional Western medicine, C = control group, T = test group.

* Time for fever reduction.

† Time for the disappearance of herpes.

‡ Incidence of adverse events (AE).

### 3.3. Quality assessment and publication bias

**Figure 2.**
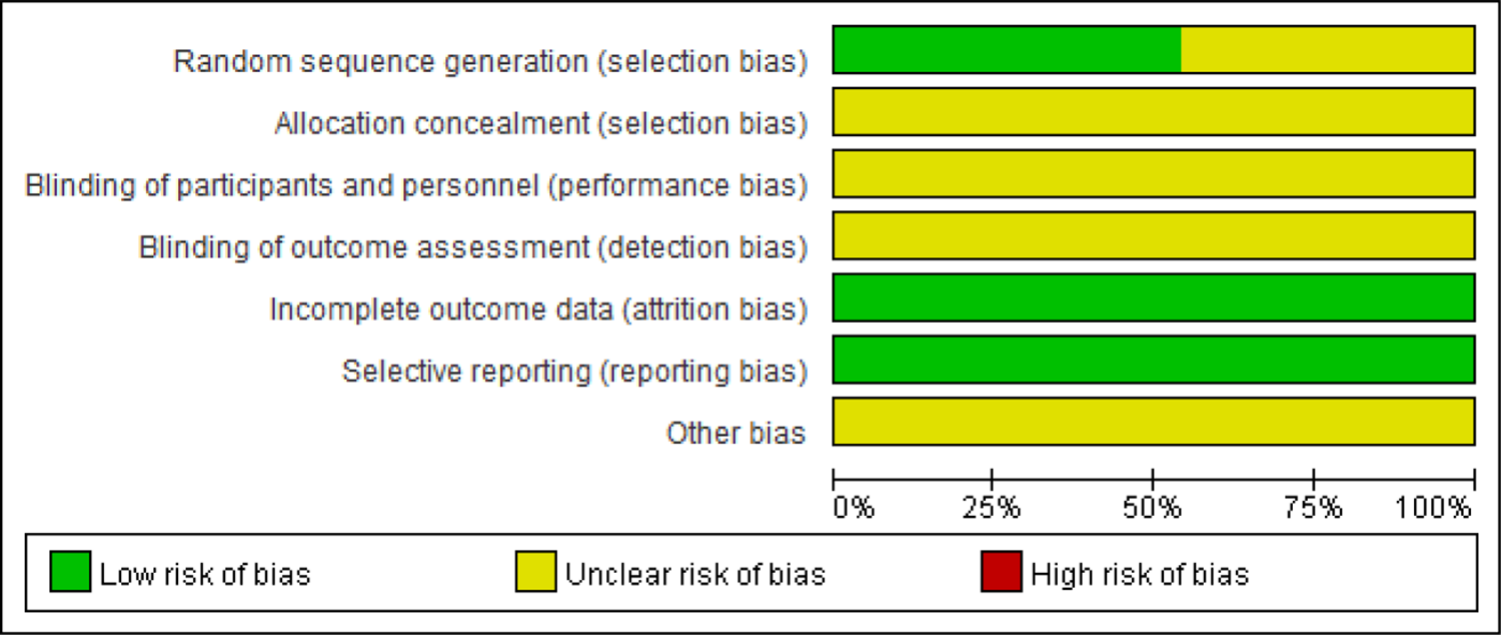
Percentage of projects with a risk of bias in the included study. Green diamonds: low risk; Red diamonds: high risk; Yellow diamonds: unclear.

Random sequence generation: Among the 33 studies evaluated as “low risk,” 32 used random number tables, and 1 employed the lottery method. The remaining 28 studies only reported “randomization” without specifying the randomization method used; this lack of detail was evaluated as an “unclear risk.” All studies had complete data, and none were selective in reporting. Therefore, all the studies were evaluated as low risk for both attrition bias and reporting bias. None mentioned the risk of bias related to blinding of implementation or outcome assessment, allocation concealment, or other sources. In the above 4 aspects, all the studies were evaluated as “unclear risk.” The proportion of items included in studies that created a risk of bias is shown in Figure 2.

### 3.4. Fever reduction time

#### 3.4.1. Network evidence figure

**Figure 3.**
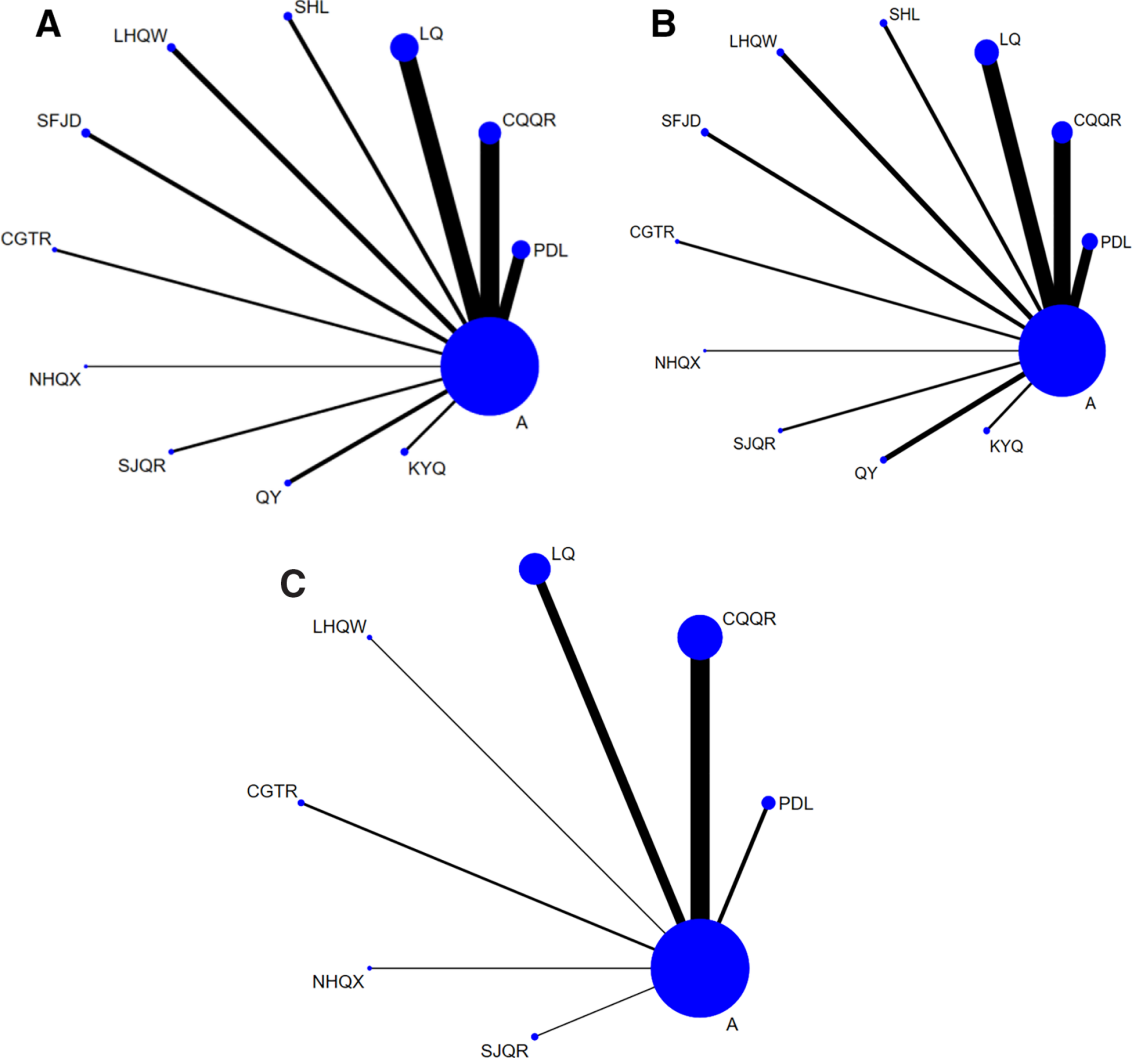
Evidence network diagram of heat reduction time (A), Herpes disappear time (B), incidence of adverse reactions (C). (A) Pudilan Anti-inflammatory Oral Liquid (PDL), Lanqin Oral Liquid (LQ), Shuanghuanglian Oral Liquid (SHL), XiaoerShuangjinqingre Oral Liquid (SJQR), Lianhuaqingwen Granules (LHQW), Xiaoer Chiqiaoqingre Granules (CQQR), XiaoerChaiguituire Granules (CGTR), XiaoerNiuhuangqingxin Powder (NHQX), Shufengjiedu Capsules (SFJD), XiaoerQingyan Granules (QY), Kouyanqing Granules (KYQ). (B) Pudilan Anti-inflammatory Oral Liquid (PDL), Lanqin Oral Liquid (LQ), Shuanghuanglian Oral Liquid (SHL), XiaoerShuangjinqingre Oral Liquid (SJQR), Lianhuaqingwen Granules (LHQW), Xiaoer Chiqiaoqingre Granules (CQQR), XiaoerChaiguituire Granules (CGTR), XiaoerNiuhuangqingxin Powder (NHQX), Shufengjiedu Capsules (SFJD), XiaoerQingyan Granules (QY), Kouyanqing Granules (KYQ). (C) Pudilan Anti-inflammatory Oral Liquid (PDL), Lanqin Oral Liquid (LQ), XiaoerShuangjinqingre Oral Liquid (SJQR), Lianhuaqingwen Granules (LHQW), Xiaoer Chiqiaoqingre Granules (CQQR), XiaoerChaiguituire Granules (CGTR), XiaoerNiuhuangqingxin Powder (NHQX).

53 studies^[8,9,11–17,19,23–25,27–61,64–68]^ reported on the duration of fever reduction involving 11 oral Chinese patent medicines. 11 direct comparisons were formed, and no closed loop formation was found. The results showed that the largest sample size (777 cases) was reported for LQ, followed by CQQR (609 cases) (Fig. 3A).

#### 3.4.2. Publication bias

**Figure 4.**
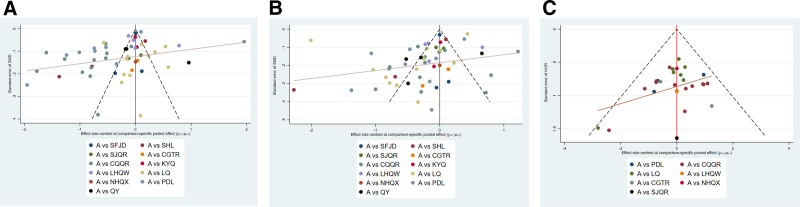
Heat reduction time (A), herpes disappear time (B), and incidence of adverse reactions (C). (A) Pudilan Anti-inflammatory Oral Liquid (PDL), Lanqin Oral Liquid (LQ), Shuanghuanglian Oral Liquid (SHL), XiaoerShuangjinqingre Oral Liquid (SJQR), Lianhuaqingwen Granules (LHQW), Xiaoer Chiqiaoqingre Granules (CQQR), XiaoerChaiguituire Granules (CGTR), XiaoerNiuhuangqingxin Powder (NHQX), Shufengjiedu Capsules (SFJD), XiaoerQingyan Granules (QY), Kouyanqing Granules (KYQ). (B) Pudilan Anti-inflammatory Oral Liquid (PDL), Lanqin Oral Liquid (LQ), Shuanghuanglian Oral Liquid (SHL), XiaoerShuangjinqingre Oral Liquid (SJQR), Lianhuaqingwen Granules (LHQW), Xiaoer Chiqiaoqingre Granules (CQQR), XiaoerChaiguituire Granules (CGTR), XiaoerNiuhuangqingxin Powder (NHQX), Shufengjiedu Capsules (SFJD), XiaoerQingyan Granules (QY), Kouyanqing Granules (KYQ). (C) Pudilan Anti-inflammatory Oral Liquid (PDL), Lanqin Oral Liquid (LQ), XiaoerShuangjinqingre Oral Liquid (SJQR), Lianhuaqingwen Granules (LHQW), Xiaoer Chiqiaoqingre Granules (CQQR), XiaoerChaiguituire Granules (CGTR), XiaoerNiuhuangqingxin Powder (NHQX).

The included studies were asymmetrically distributed around the vertical line, indicating possible publication bias and small sample effects (Fig. 4A).

#### 3.4.3. Network meta-analysis

A total of 66 two-by-two comparisons were generated. Among these, 15 showed statistically significant differences. Compared with conventional Western medicine treatment, the combined therapies of SJQR, LHQW, PDL, LQ, QY, SHL, and CQQR reduced fever duration more quickly. Specifically, SJQR was superior to LQ, SHL, CGTR, CQQR, KYQ, and SFJD. Additionally, LHQW outperformed CQQR and SFJD. The differences between the remaining interventions were not statistically significant when compared two-by-two (Table 2).

**Table 2:** Network meta-analysis for fever reduction time (odds ratio, 95% CI).

SJQR + A											
−0.50 (−1.57, 0.58)	**LHQW + A**										
−0.79 (−1.78, 0.19)	−0.29 (−1.05, 0.46)	**PDL + A**									
−0.94 (−2.47, 0.59)	−0.44 (−1.84, 0.96)	−0.15 (−1.48, 1.18)	**NHQX + A**								
**−0.99 (−1.95, −0.04**)	−0.49 (−1.21, 0.22)	−0.20 (−0.77, 0.37)	−0.05 (−1.36, 1.26)	**LQ + A**							
−1.01 (−2.14, 0.13)	−0.51 (−1.45, 0.44)	−0.21 (−1.06, 0.63)	−0.07 (−1.51, 1.38)	−0.02 (−0.82, 0.79)	**QY + A**						
**−1.18 (−2.32, −0.04**)	−0.68 (−1.63, 0.27)	−0.39 (−1.24, 0.46)	−0.24 (−1.69, 1.21)	−0.19 (−1.00, 0.63)	−0.17 (−1.19, 0.85)	**SHL + A**					
**−1.29 (−2.55, −0.04**)	−0.79 (−1.88, 0.29)	−0.50 (−1.49, 0.50)	−0.35 (−1.89, 1.19)	−0.30 (−1.26, 0.67)	−0.28 (−1.43, 0.86)	−0.11 (−1.26, 1.04)	**CGTR + A**				
**−1.29 (−2.24, −0.34**)	**−0.79 (−1.50, −0.08**)	−0.49 (−1.06, 0.07)	−0.35 (−1.65, 0.96)	−0.30 (−0.80, 0.21)	−0.28 (−1.08, 0.52)	−0.11 (−0.92, 0.70)	0.00 (−0.96, 0.96)	**CQQR + A**			
**−1.36 (−2.60, −0.13**)	−0.87 (−1.93, 0.20)	−0.57 (−1.55, 0.40)	−0.42 (−1.95, 1.10)	−0.37 (−1.32, 0.57)	−0.36 (−1.48, 0.77)	−0.18 (−1.32, 0.95)	−0.07 (−1.32, 1.17)	−0.08 (−1.01, 0.86)	**KYQ + A**		
**−1.51 (−2.65, −0.36**)	**−1.01 (−1.96, −0.06**)	−0.71 (−1.56, 0.14)	−0.57 (−2.02, 0.88)	−0.51 (−1.33, 0.30)	−0.50 (−1.52, 0.52)	−0.33 (−1.36, 0.70)	−0.22 (−1.37, 0.94)	−0.22 (−1.03, 0.59)	−0.14 (−1.27, 0.99)	**SFJD + A**	
**−2.14 (−3.02, −1.26**)	**−1.64 (−2.26, −1.03**)	**−1.35 (−1.79, −0.91**)	−1.20 (−2.46, 0.06)	**−1.15 (−1.51, −0.78**)	**−1.13 (−1.85, −0.42**)	**−0.96 (−1.69, −0.23**)	−0.85 (−1.74, 0.04)	**−0.85 (−1.20, −0.50**)	−0.78 (−1.64, 0.09)	−0.63 (−1.36, 0.09)	**A**

Bold values indicate a statistically significant difference between interventions.

A = conventional Western medicine, CGTR = XiaoerChaiguituire Granules, CQQR = XiaoerChiqiaoqingre Granules, KYQ = Kouyanqing Granules, LHQW = Lianhuaqingwen Granules, LQ = Lanqin Oral Liquid, NHQX = XiaoerNiuhuangqingxin Powder, PDL = Pudilan Anti-inflammatory Oral Liquid, QY = XiaoerQingyan Granules, SFJD = Shufengjiedu Capsules, SHL = Shuanghuanglian Oral Liquid, SJQR = XiaoerShuangjinqingre Oral Liquid.

#### 3.4.4. Ranking of the results of reticulated meta-analysis

The time to reduce fever was evaluated using a ranking probability table. The treatments were ranked from best to worst as follows: SJQR, LHQW, PDL, NHQX, LQ, QY, SHL, CGTR, CQQR, KYQ, and SFJD (Table 3).

**Table 3:** SUCRA ranking for outcome indicator improved by oral Chinese patent medicine combined with conventional treatment.

Intervention	Heat reduction time		Herpes disappear time		Incidence of adverse reactions	
	SUCRA%	Rank	SUCRA%	Rank	SUCRA%	Rank
PDL + A	70.9	3	79.9	3	68.2	2
CQQR + A	35.3	9	70.7	4	75.1	1
LQ + A	56.9	5	37.1	8	67.5	3
SHL + A	43.9	7	67.6	5		
LHQW + A	84.0	2	53.9	7	36.6	6
SFJD + A	26.2	11	23.2	10		
CGTR + A	38.4	8	18.1	11	61.5	4
NHQX + A	57.6	4	57.9	6	25.9	7
SJQR + A	95.4	1	81.4	1	24.6	8
QY + A	55.3	6	29.0	9		
KYQ + A	34.7	10	80.6	2		
A	1.3	12	0.5	12	40.6	5

A = conventional Western medicine, CGTR = XiaoerChaiguituire Granules, CQQR = XiaoerChiqiaoqingre Granules, KYQ = Kouyanqing Granules, LHQW = Lianhuaqingwen Granules, LQ = Lanqin Oral Liquid, NHQX = XiaoerNiuhuangqingxin Powder, PDL = Pudilan Anti-inflammatory Oral Liquid, QY = XiaoerQingyan Granules, SFJD = Shufengjiedu Capsules, SHL = Shuanghuanglian Oral Liquid, SJQR = XiaoerShuangjinqingre Oral Liquid, SUCRA = the cumulative ranking curve.

#### 3.4.5. Inconsistency test

**Figure 5.**
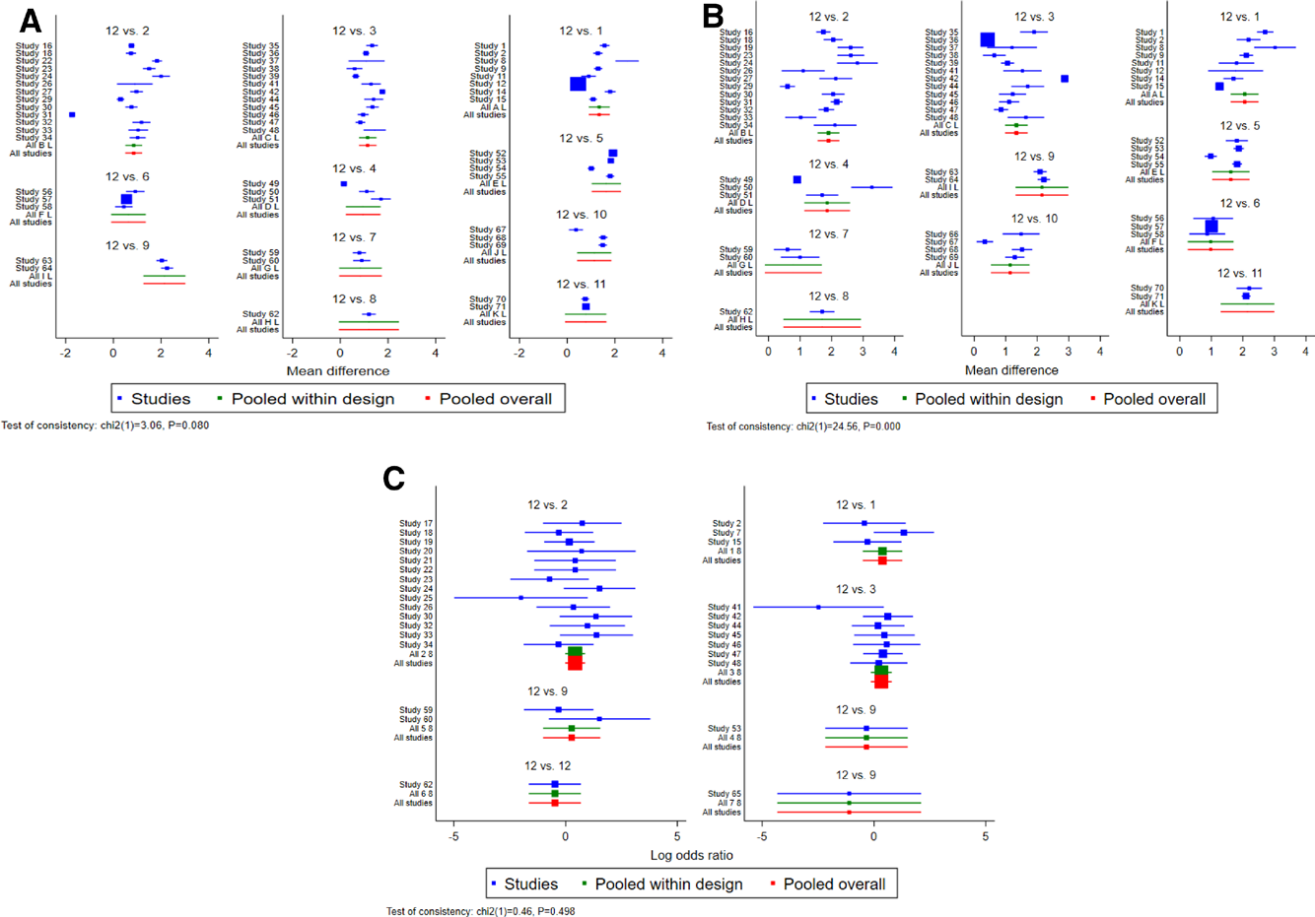
Heat reduction time (A), herpes disappear time (B), and incidence of adverse reactions (C). 1 = PDL + A;2 = CQQR + A;3 = LQ + A;4 = SHL + A;5 = LHQW + A;6 = SFJD + A;7 = CGTR + A;8 = NHQX + A;9 = SJQR + A;10 = QY + A;11 = KYQ + A;12 = A. CGTR = XiaoerChaiguituire Granules, CQQR = XiaoerChiqiaoqingre Granules, KYQ = Kouyanqing Granules, LHQW = Lianhuaqingwen Granules, LQ = Lanqin Oral Liquid, NHQX = XiaoerNiuhuangqingxin Powder, PDL = Pudilan Anti-inflammatory Oral Liquid, QY = XiaoerQingyan Granules, SFJD = Shufengjiedu Capsules, SHL = Shuanghuanglian Oral Liquid, SJQR = XiaoerShuangjinqingre Oral Liquid.

Through the overall inconsistency test, there was no significant difference in the fever reduction time (*P* = .08 > 0.05) between the consistency and inconsistency models. Therefore, the individual studies could be combined for comparison (Fig. 5A).

### 3.5. Herpes remission time

#### 3.5.1. Network evidence graph

54 studies^[8,9,11–17,19,20,24,25,27–61,63–68]^ reported the time to herpes remission involving 11 oral Chinese patent medicines. 11 direct comparisons were formed, and no closed loop formation was found. The results showed that the largest sample size (777 cases) was reported for LQ, followed by CQQR (658 cases) (Fig. 3B).

#### 3.5.2. Publication bias

The included studies were not symmetrically distributed on both sides of the central line, suggesting some publication bias. Some studies were distributed below the inverted funnel plot, suggesting the possibility of a small-study effect (Fig. 4B).

#### 3.5.3. Network meta-analysis

A total of 66 two-by-two comparisons were generated, of which 23 showed statistically significant differences. The combination of the 11 oral Chinese patent medicines studied resulted in a shorter time to herpes lesion regression compared with conventional treatment using Western medicine alone. Additionally, SJQR demonstrated greater efficacy than SFJD and CGTR; KYQ was more effective than SFJD and CGTR; PDL outperformed LQ, QY, SFJD, and CGTR; and CQQR showed superior efficacy compared to LQ, QY, SFJD, and CGTR. The differences between the remaining interventions were not statistically significant when compared between any 2 (Table 4).

**Table 4:** Network meta-analysis for herpes remission time (odds ratio, 95% CI).

SJQR + A											
−0.00 (−1.19, 1.19)	**KYQ + A**										
−0.09 (−1.04, 0.85)	−0.09 (−1.05, 0.87)	**PDL + A**									
−0.25 (−1.15, 0.66)	−0.25 (−1.16, 0.67)	−0.15 (−0.71, 0.41)	**CQQR + A**								
−0.29 (−1.40, 0.82)	−0.29 (−1.40, 0.83)	−0.20 (−1.04, 0.65)	−0.04 (−0.84, 0.76)	**SHL + A**							
−0.45 (−1.94, 1.04)	−0.45 (−1.94, 1.05)	−0.36 (−1.66, 0.95)	−0.20 (−1.48, 1.07)	−0.16 (−1.59, 1.27)	**NHQX + A**						
−0.53 (−1.56, 0.49)	−0.53 (−1.56, 0.51)	−0.44 (−1.18, 0.30)	−0.28 (−0.97, 0.40)	−0.24 (−1.18, 0.69)	−0.08 (−1.45, 1.28)	**LHQW + A**					
−0.81 (−1.72, 0.10)	−0.81 (−1.73, 0.11)	**−0.72 (−1.29, −0.15**)	**−0.56 (−1.06, −0.07**)	−0.52 (−1.33, 0.29)	−0.36 (−1.64, 0.92)	−0.28 (−0.97, 0.41)	**LQ + A**				
−1.01 (−2.04, 0.03)	−1.00 (−2.05, 0.04)	**−0.91 (−1.67, −0.16**)	**−0.76 (−1.46, −0.06**)	−0.72 (−1.67, 0.23)	−0.56 (−1.93, 0.82)	−0.48 (−1.33, 0.38)	−0.20 (−0.90, 0.51)	**QY + A**			
**−1.17 (−2.28, −0.06**)	**−1.17 (−2.28, −0.05**)	**−1.08 (−1.92, −0.23**)	**−0.92 (−1.72, −0.12**)	−0.88 (−1.91, 0.15)	−0.72 (−2.14, 0.71)	−0.64 (−1.57, 0.30)	−0.36 (−1.17, 0.45)	−0.16 (−1.11, 0.79)	**SFJD + A**		
**−1.36 (−2.59, −0.13**)	**−1.36 (−2.60, −0.12**)	**−1.27 (−2.27, −0.26**)	**−1.11 (−2.08, −0.15**)	−1.07 (−2.23, 0.09)	−0.91 (−2.44, 0.61)	−0.83 (−1.91, 0.25)	−0.55 (−1.52, 0.42)	−0.35 (−1.45, 0.74)	−0.19 (−1.35, 0.97)	**CGTR + A**	
**−2.15 (−2.99, −1.31**)	**−2.15 (−3.00, −1.30**)	**−2.06 (−2.50, −1.62**)	**−1.90 (−2.25, −1.56**)	**−1.86 (−2.59, −1.14**)	**−1.70 (−2.93, −0.47**)	**−1.62 (−2.21, −1.03**)	**−1.34 (−1.70, −0.98**)	**−1.14 (−1.76, −0.53**)	**−0.98 (−1.71, −0.26**)	**−0.79 (−1.69, 0.11**)	**A**

Bold values indicate a statistically significant difference between interventions.

A = conventional Western medicine, CGTR = XiaoerChaiguituire Granules, CQQR = XiaoerChiqiaoqingre Granules, KYQ = Kouyanqing Granules, LHQW = Lianhuaqingwen Granules, LQ = Lanqin Oral Liquid, NHQX = XiaoerNiuhuangqingxin Powder, PDL = Pudilan Anti-inflammatory Oral Liquid, QY = XiaoerQingyan Granules, SFJD = Shufengjiedu Capsules, SHL = Shuanghuanglian Oral Liquid, SJQR = XiaoerShuangjinqingre Oral Liquid.

#### 3.5.4. Ranking of results by network meta-analysis

Herpes regression time was evaluated by ranking the probability table from best to worst as SJQR, KYQ, PDL, CQQR, SHL, NHQX, LHQW, LQ, QY, SFJD, and CGTR (Table 3).

#### 3.5.5. Inconsistency test

Through the overall inconsistency test, we found that for the herpes remission time (*P* < .01), which indicates a large degree of heterogeneity among different intervention measures. This may have an impact on the research results. Therefore, we used the node-splitting method to conduct a local inconsistency test for the herpes remission time. The results showed that there was no statistically significant difference between the direct and indirect comparison results at each split node (*P* > .05), meaning that the comparison of any 2 intervention measures was consistent. Through analysis, the cause of the overall inconsistency may stem from the fact that different studies did not have specific measurement criteria for the herpes remission time, resulting in significant differences in the study design (Fig. 5B).

### 3.6. AE incidence

#### 3.6.1. Network evidence map

The incidence of AE was reported in 29 studies^[9,10,16,18–27,30,32–34,40–46,51,57–59,62]^ involving 7 oral Chinese patent medicines. 7 direct comparisons were formed, and there was no closed loop formation. The results showed the largest number of studies (14 RCTs) and the largest sample size (756 cases) of CQQR combined with conventional treatment with Western medicine (Fig. 3C).

#### 3.6.2. Publication bias

The large sample of studies was essentially symmetrically distributed on both sides of the vertical line, suggesting a low likelihood of publication bias (Fig. 4C).

#### 3.6.3. Network meta-analysis

A total of 28 pairwise comparisons were generated for the 8 interventions, and none of the differences were statistically significant (Table 5).

**Table 5:** Network meta-analysis for AE incidence (odds ratio, 95% CI).

CQQR + A							
0.95 (0.35, 2.57)	**PDL + A**						
0.91 (0.47, 1.73)	0.95 (0.35, 2.59)	**LQ + A**					
0.86 (0.22, 3.34)	0.90 (0.19, 4.27)	0.95 (0.24, 3.71)	**CGTR + A**				
0.65 (0.42, 1.02)	0.69 (0.28, 1.66)	0.72 (0.45, 1.16)	0.76 (0.21, 2.74)	**A**			
0.47 (0.07, 3.11)	0.49 (0.06, 3.77)	0.52 (0.08, 3.45)	0.54 (0.06, 5.12)	0.72 (0.11, 4.50)	**LHQW + A**		
0.41 (0.12, 1.41)	0.42 (0.10, 1.83)	0.45 (0.13, 1.57)	0.47 (0.08, 2.65)	0.62 (0.19, 1.98)	0.87 (0.10, 7.62)	**NHQX + A**	
0.22 (0.01, 5.53)	0.23 (0.01, 6.33)	0.24 (0.01, 6.13)	0.25 (0.01, 7.98)	0.33 (0.01, 8.20)	0.46 (0.01, 18.69)	0.53 (0.02, 16.24)	**SJQR + A**

Bold values indicate a statistically significant difference between interventions.

A = conventional Western medicine, AE = adverse events, CGTR = XiaoerChaiguituire Granules, CQQR = XiaoerChiqiaoqingre Granules, LHQW = Lianhuaqingwen Granules, LQ = Lanqin Oral Liquid, NHQX = XiaoerNiuhuangqingxin Powder, PDL = Pudilan Anti-inflammatory Oral Liquid, SJQR = XiaoerShuangjinqingre Oral Liquid.

#### 3.6.4. Ranking of the results of network meta-analysis

The incidence of AE was evaluated by ranking the probability table from best to worst as CQQR, PDL, LQ, CGTR, LHQW, NHQX, and SJQR (Table 3).

#### 3.6.5. Inconsistency test

The overall inconsistency test revealed that for the incidence of adverse reactions (*P* = .498 > .05), there was no significant difference between the consistency and inconsistency models. Thus, the individual studies could be combined for comparison satisfactorily (Fig. 5C).

## 4. Discussion

This network meta-analysis showed that combining oral Chinese patent medicine with conventional Western medicine improves efficacy in treating herpangina. However, the advantages vary depending on the distinct characteristics of Chinese patent medicines.

Regarding clinical efficacy, SJQR has a significant advantage in reducing fever and has the best effect in eliminating herpes. The medicine is composed of 16 herbs. Among them, honeysuckle, dandelion, indigo woad leaf, indigo woad root, bupleurum, and other herbs are known in traditional Chinese medicine to help reduce inflammation and eliminate toxins. Schizonepeta can relieve the sweat and dispel the wind. Platycodon grandiflorum and stiff silkworms can alleviate sore throat. The compatibility of the above ingredients makes SJQR anti-inflammatory, antibacterial, detumescent, analgesic, and other effects.^[61,62]^ Pharmacological studies showed that many herbs in the formula, such as Bupleurum, Isatis leaf, honeysuckle, Isatis root, and Schizonepeta, have demonstrated antiviral properties. Additionally, some in vitro studies showed that SJQR could inhibit the replication of EV71 and Coxsackie B3 viruses.^[69]^

PDL was ranked in the top 3 for both efficacy outcome indicators, showing that the drug effectively relieved the main symptoms of herpangina. PDL is mainly composed of a formula of 4 cold and bitter herbs with clearing and detoxifying effects. The dandelion has a diuretic effect; the Bunge corydalis herb clears heat and eliminates nodules; indigo woad root cools the blood and benefits the throat; and Baikal skullcap root dries dampness and relieves fire.^[70]^ Studies showed that the drug significantly inhibited the increased body temperature induced by dried yeast in rats. This indicates its superior antipyretic effect.^[71]^ Network pharmacological studies suggest that the effect of PDL on herpangina may be related to the modulation of heat shock protein 90α (HSP90AA1) by several of its active ingredients.^[72]^

LHQW is second only to SJQR in reducing fever and is widely used to treat viral infectious diseases of the respiratory system. It has especially played an important role during influenza and novel coronavirus pneumonia epidemics in recent years.^[73]^ LHQW mainly contains forsythia suspensa, honeysuckle, stir-fried ephedra, gypsum, indigo woad root, and 8 other herbs. These components work together to clear the plague, detoxify, promote lung function, and drain heat.^[74]^ Among them, honeysuckle, forsythia suspensa, and indigo woad root could not only clear heat in internal organs but also resolve the heat toxicity in the blood.^[50]^ Stir-fried ephedra can release sweat and relieve symptoms, and menthol can remove wind and clear heat.^[52]^ These are all related to the good antipyretic effect of LHQW. In addition, modern pharmacological studies showed that LHQW has antiviral, antibacterial, anti-inflammatory, and antitumor pharmacological effects.^[74]^ Some studies showed that LHQW has inhibitory effects on EV71 and Coxsackie B4 viruses.^[73]^ However, LHQW ranked 7th out of 11 varieties in terms of herpes regression and is recommended for children with fever as the main symptom.

KYQ is second only to SJQR in eliminating herpes. Among this medicine’s main ingredients, asparagus and liriope contribute to nourishing yin and engendering liquid. Liriope can also improve cellular immune function. Figwort has the effect of anti-inflammatory, antibacterial, immune enhancing activity, and antinociception. In addition, honeysuckles also have anti-inflammatory and immunomodulatory effects.^[67]^ Modern pharmacological studies have shown that the drug can inhibit the growth and reproduction of various bacteria in vitro.^[75]^ It also improves the child’s immunity, and promote rapid healing of herpes wounds.^[68]^ However, the drug ranked 10th in reducing fever, which appears to have no significant advantage. It’s considered to be related to the lack of herbs in its formula for wind-coursing and heat-clearing. The study results also matched the indications in the KYQ instructions.

Regarding safety, no statistically significant difference was found in either the direct comparison of each combination regimen with conventional Western drugs alone or the indirect comparison between the combination regimens. This suggests that the oral Chinese patent medicines included in the study are safe.

Advantages and limitations of this study: There was less direct RCT between each oral Chinese patent medicine. In this study, the efficacy and safety of 11 commonly used oral Chinese patent medicines were indirectly compared using mesh meta-analysis. The outcome measures included the most important symptoms of herpes pharyngitis, specifically fever and herpetic lesions. The efficacy of treatments for these symptoms was ranked accordingly. This ranking provided a reference for managing children with different clinical presentations. However, there are still some limitations in the study. Firstly, the outcome “herpes regression time” is potentially subjective. The outcome “herpes regression time” is defined as the time from treatment initiation until the complete regression or disappearance of oral herpes. None of the included primary studies provided detailed descriptions of the measurement methodology for this outcome (e.g., who performed the assessment, assessment frequency, specific visual criteria). However, all studies explicitly used “complete regression” or “disappearance” of herpes as the endpoint, rather than “improvement.” Using “complete disappearance” as an endpoint likely provides greater objectivity and consistency in visual assessment than “improvement.” However, potential bias from subjective assessment cannot be entirely ruled out. Secondly, all studies did not report on the implementation of blinding of assessors. We cannot determine whether these studies were blinded to the assessors. The absence of blinding may influence their judgment about whether herpes has completely disappeared, especially when the condition is close to resolution. This may be lead to detection or assessment bias. This potential source of bias should be fully considered in interpreting the results. Thirdly, research on NHQX is limited to only 1 study, on KYQ to only 2 studies, and on CGTR also to only 2 studies. Moreover, these studies generally had small sample sizes. These limitations constrain the reliability of their comparative rankings. We supplemented a sensitivity analysis to compare whether the number of included studies was less than 3 or the intervention measures with a relatively small sample size would cause changes in the result ranking. Actually, after excluding these studies, the new result ranking showed no change compared with the original result (Tables 6–8). This shows that the results of this study have certain reference value, but clinicians should interpret the rankings of these 3 drugs cautiously. Finally, 28 studies had unclear randomization methods. Unclear randomization may cause baseline imbalances, which distort effect sizes and reduce SUCRA’s reliability in ranking interventions with small differences in efficacy. Therefore, we conducted a sensitivity analysis and excluded 2 studies^[24,37]^ with unknown randomization methods. SUCRA ranking did not change significantly (Tables 9–11), indicating that the core conclusions were robust to the risk of randomization bias.

**Table 10:** Sensitivity analysis regarding unclear randomization of herpes remission time.

PDL + A	0.21 (−0.38, 0.81)	0.19 (−0.69, 1.07)	0.44 (−0.33, 1.21)	0.71 (0.11, 1.31)	0.91 (0.13, 1.70)	1.08 (0.19, 1.96)	2.06 (1.60, 2.52)
−0.21 (−0.81, 0.38)	**CQQR + A**	−0.02 (−0.87, 0.82)	0.22 (−0.50, 0.95)	0.49 (−0.04, 1.03)	0.70 (−0.04, 1.44)	0.86 (0.02, 1.70)	1.84 (1.47, 2.22)
−0.19 (−1.07, 0.69)	0.02 (−0.82, 0.87)	**SHL + A**	0.25 (−0.72, 1.22)	0.52 (−0.33, 1.36)	0.72 (−0.26, 1.71)	0.89 (−0.18, 1.95)	1.87 (1.12, 2.62)
−0.44 (−1.21, 0.33)	−0.22 (−0.95, 0.50)	−0.25 (−1.22, 0.72)	**LHQW + A**	0.27 (−0.46, 1.00)	0.47 (−0.41, 1.36)	0.64 (−0.34, 1.61)	1.62 (1.00, 2.24)
−0.71 (−1.31, −0.11)	−0.49 (−1.03, 0.04)	−0.52 (−1.36, 0.33)	−0.27 (−1.00, 0.46)	**LQ + A**	0.21 (−0.54, 0.95)	0.37 (−0.48, 1.21)	1.35 (0.96, 1.73)
−0.91 (−1.70, −0.13)	−0.70 (−1.44, 0.04)	−0.72 (−1.71, 0.26)	−0.47 (−1.36, 0.41)	−0.21 (−0.95, 0.54)	**QY + A**	0.16 (−0.82, 1.15)	1.14 (0.51, 1.78)
−1.08 (−1.96, −0.19)	−0.86 (−1.70, −0.02)	−0.89 (−1.95, 0.18)	−0.64 (−1.61, 0.34)	−0.37 (−1.21, 0.48)	−0.16 (−1.15, 0.82)	**SFJD + A**	0.98 (0.23, 1.73)
−2.06 (−2.52, −1.60)	−1.84 (−2.22, −1.47)	−1.87 (−2.62, −1.12)	−1.62 (−2.24, −1.00)	−1.35 (−1.73, −0.96)	−1.14 (−1.78, −0.51)	−0.98 (−1.73, −0.23)	**A**

Bold values indicate a statistically significant difference between interventions.

A = conventional Western medicine, CQQR = XiaoerChiqiaoqingre Granules, LHQW = Lianhuaqingwen Granules, LQ = Lanqin Oral Liquid, PDL = Pudilan Anti-inflammatory Oral Liquid, QY = XiaoerQingyan Granules, SFJD = Shufengjiedu Capsules, SHL = Shuanghuanglian Oral Liquid.

**Table 11:** Sensitivity analysis regarding unclear randomization of AE incidence.

CQQR + A	1.13 (0.42, 3.07)	1.19 (0.62, 2.31)	1.65 (1.04, 2.62)
0.88 (0.33, 2.39)	**PDL + A**	1.05 (0.39, 2.87)	1.46 (0.60, 3.53)
0.84 (0.43, 1.62)	0.95 (0.35, 2.59)	**LQ + A**	1.39 (0.87, 2.22)
0.60 (0.38, 0.96)	0.69 (0.28, 1.66)	0.72 (0.45, 1.16)	**A**

Bold values indicate a statistically significant difference between interventions.

A = conventional Western medicine, AE = adverse events, CQQR = XiaoerChiqiaoqingre Granules, PDL = Pudilan Anti-inflammatory Oral Liquid, LQ = Lanqin Oral Liquid.

**Table 6:** Sensitivity analysis after excluding studies with small sample sizes of fever reduction time.

LHQW + A	0.29 (−0.49, 1.08)	0.49 (−0.25, 1.23)	0.51 (−0.47, 1.49)	0.68 (−0.31, 1.67)	0.79 (0.05, 1.52)	1.01 (0.02, 1.99)	1.64 (1.00, 2.28)
−0.29 (−1.08, 0.49)	**PDL + A**	0.20 (−0.39, 0.79)	0.22 (−0.66, 1.09)	0.39 (−0.50, 1.27)	0.49 (−0.09, 1.08)	0.71 (−0.17, 1.60)	1.35 (0.89, 1.80)
−0.49 (−1.23, 0.25)	−0.20 (−0.79, 0.39)	**LQ + A**	0.02 (−0.82, 0.85)	0.19 (−0.66, 1.03)	0.30 (−0.23, 0.82)	0.51 (−0.33, 1.36)	1.15 (0.77, 1.53)
−0.51 (−1.49, 0.47)	−0.22 (−1.09, 0.66)	−0.02 (−0.85, 0.82)	**QY + A**	0.17 (−0.89, 1.23)	0.28 (−0.55, 1.11)	0.50 (−0.56, 1.56)	1.13 (0.39, 1.88)
−0.68 (−1.67, 0.31)	−0.39 (−1.27, 0.50)	−0.19 (−1.03, 0.66)	−0.17 (−1.23, 0.89)	**SHL + A**	0.11 (−0.73, 0.95)	0.33 (−0.74, 1.39)	0.96 (0.21, 1.72)
−0.79 (−1.52, −0.05)	−0.49 (−1.08, 0.09)	−0.30 (−0.82, 0.23)	−0.28 (−1.11, 0.55)	−0.11 (−0.95, 0.73)	**CQQR + A**	0.22 (−0.62, 1.06)	0.85 (0.49, 1.22)
−1.01 (−1.99, −0.02)	−0.71 (−1.60, 0.17)	−0.51 (−1.36, 0.33)	−0.50 (−1.56, 0.56)	−0.33 (−1.39, 0.74)	−0.22 (−1.06, 0.62)	**SFJD + A**	0.63 (−0.12, 1.39)
−1.64 (−2.28, −1.00)	−1.35 (−1.80, −0.89)	−1.15 (−1.53, −0.77)	−1.13 (−1.88, −0.39)	−0.96 (−1.72, −0.21)	−0.85 (−1.22, −0.49)	−0.63 (−1.39, 0.12)	**A**

Bold values indicate a statistically significant difference between interventions.

A = conventional Western medicine, CQQR = XiaoerChiqiaoqingre Granules, LHQW = Lianhuaqingwen Granules, LQ = Lanqin Oral Liquid, PDL = Pudilan Anti-inflammatory Oral Liquid, QY = XiaoerQingyan Granules, SFJD = Shufengjiedu Capsules, SHL = Shuanghuanglian Oral Liquid.

**Table 7:** Sensitivity analysis after excluding studies with small sample sizes of herpes remission time.

PDL + A	0.16 (−0.42, 0.73)	0.19 (−0.69, 1.07)	0.44 (−0.33, 1.20)	0.72 (0.13, 1.31)	0.91 (0.13, 1.69)	1.08 (0.20, 1.95)	2.06 (1.60, 2.51)
−0.16 (−0.73, 0.42)	**CQQR + A**	0.04 (−0.79, 0.86)	0.28 (−0.43, 0.99)	0.56 (0.05, 1.08)	0.76 (0.03, 1.49)	0.92 (0.09, 1.75)	1.90 (1.55, 2.26)
−0.19 (−1.07, 0.69)	−0.04 (−0.86, 0.79)	**SHL + A**	0.25 (−0.72, 1.22)	0.53 (−0.31, 1.36)	0.72 (−0.26, 1.70)	0.89 (−0.17, 1.95)	1.87 (1.12, 2.62)
−0.44 (−1.20, 0.33)	−0.28 (−0.99, 0.43)	−0.25 (−1.22, 0.72)	**LHQW + A**	0.28 (−0.44, 1.00)	0.47 (−0.41, 1.36)	0.64 (−0.33, 1.61)	1.62 (1.00, 2.23)
−0.72 (−1.31, −0.13)	−0.56 (−1.08, −0.05)	−0.53 (−1.36, 0.31)	−0.28 (−1.00, 0.44)	**LQ + A**	0.20 (−0.54, 0.93)	0.36 (−0.48, 1.19)	1.34 (0.97, 1.71)
−0.91 (−1.69, −0.13)	−0.76 (−1.49, −0.03)	−0.72 (−1.70, 0.26)	−0.47 (−1.36, 0.41)	−0.20 (−0.93, 0.54)	**QY + A**	0.16 (−0.82, 1.14)	1.14 (0.51, 1.78)
−1.08 (−1.95, −0.20)	−0.92 (−1.75, −0.09)	−0.89 (−1.95, 0.17)	−0.64 (−1.61, 0.33)	−0.36 (−1.19, 0.48)	−0.16 (−1.14, 0.82)	**SFJD + A**	0.98 (0.23, 1.73)
−2.06 (−2.51, −1.60)	−1.90 (−2.26, −1.55)	−1.87 (−2.62, −1.12)	−1.62 (−2.23, −1.00)	−1.34 (−1.71, −0.97)	−1.14 (−1.78, −0.51)	−0.98 (−1.73, −0.23)	**A**

Bold values indicate a statistically significant difference between interventions.

A = conventional Western medicine, CQQR = XiaoerChiqiaoqingre Granules, LHQW = Lianhuaqingwen Granules, LQ = Lanqin Oral Liquid, PDL = Pudilan Anti-inflammatory Oral Liquid, QY = XiaoerQingyan Granules, SFJD = Shufengjiedu Capsules, SHL = Shuanghuanglian Oral Liquid.

**Table 8:** Sensitivity analysis after excluding studies with small sample sizes of AE incidence.

CQQR + A	1.05 (0.39, 2.82)	1.10 (0.58, 2.11)	1.53 (0.98, 2.39)
0.95 (0.35, 2.57)	**PDL + A**	1.05 (0.39, 2.87)	1.46 (0.60, 3.53)
0.91 (0.47, 1.73)	0.95 (0.35, 2.59)	**LQ + A**	1.39 (0.87, 2.22)
0.65 (0.42, 1.02)	0.69 (0.28, 1.66)	0.72 (0.45, 1.16)	**A**

Bold values indicate a statistically significant difference between interventions.

A = conventional Western medicine, AE = adverse events, CQQR = XiaoerChiqiaoqingre Granules.

**Table 9:** Sensitivity analysis regarding unclear randomization of fever reduction time.

LHQW + A	0.29 (−0.50, 1.09)	0.49 (−0.27, 1.24)	0.51 (−0.48, 1.50)	0.68 (−0.32, 1.68)	0.84 (0.09, 1.59)	1.01 (0.01, 2.00)	1.64 (1.00, 2.28)
−0.29 (−1.09, 0.50)	**PDL + A**	0.20 (−0.41, 0.80)	0.22 (−0.66, 1.10)	0.39 (−0.50, 1.28)	0.55 (−0.05, 1.15)	0.71 (−0.18, 1.60)	1.35 (0.89, 1.81)
−0.49 (−1.24, 0.27)	−0.20 (−0.80, 0.41)	**LQ + A**	0.02 (−0.83, 0.87)	0.19 (−0.67, 1.05)	0.35 (−0.20, 0.90)	0.52 (−0.34, 1.38)	1.15 (0.76, 1.55)
−0.51 (−1.50, 0.48)	−0.22 (−1.10, 0.66)	−0.02 (−0.87, 0.83)	**QY + A**	0.17 (−0.90, 1.24)	0.33 (−0.51, 1.18)	0.50 (−0.57, 1.57)	1.13 (0.38, 1.88)
−0.68 (−1.68, 0.32)	−0.39 (−1.28, 0.50)	−0.19 (−1.05, 0.67)	−0.17 (−1.24, 0.90)	**SHL + A**	0.16 (−0.69, 1.01)	0.33 (−0.75, 1.40)	0.96 (0.20, 1.72)
−0.84 (−1.59, −0.09)	−0.55 (−1.15, 0.05)	−0.35 (−0.90, 0.20)	−0.33 (−1.18, 0.51)	−0.16 (−1.01, 0.69)	**CQQR + A**	0.16 (−0.69, 1.02)	0.80 (0.42, 1.18)
−1.01 (−2.00, −0.01)	−0.71 (−1.60, 0.18)	−0.52 (−1.38, 0.34)	−0.50 (−1.57, 0.57)	−0.33 (−1.40, 0.75)	−0.16 (−1.02, 0.69)	**SFJD + A**	0.63 (−0.13, 1.39)
−1.64 (−2.28, −1.00)	−1.35 (−1.81, −0.89)	−1.15 (−1.55, −0.76)	−1.13 (−1.88, −0.38)	−0.96 (−1.72, −0.20)	−0.80 (−1.18, −0.42)	−0.63 (−1.39, 0.13)	**A**

Bold values indicate a statistically significant difference between interventions.

A = conventional Western medicine, CQQR = XiaoerChiqiaoqingre Granules, LHQW = Lianhuaqingwen Granules, LQ = Lanqin Oral Liquid, PDL = Pudilan Anti-inflammatory Oral Liquid, QY = XiaoerQingyan Granules, SFJD = Shufengjiedu Capsules, SHL = Shuanghuanglian Oral Liquid.

## 5. Conclusion

In summary, for children with herpangina, adding an oral Chinese patent medicine to conventional Western medical treatment can improve efficacy to some extent without increasing adverse reactions. Among them, SJQR and PDL have better effects for treating fever and herpes. They are recommended as the best treatment option. In addition, LHQW is more effective in reducing fever but less effective in reducing herpes. It is recommended for children with fever as the main symptom. KYQ is more effective in reducing herpes but less effective in reducing fever. It can be used for children with oropharyngeal herpes as the main symptom. However, the limited evidence for KYQ (2 trials) precludes definitive conclusions on its efficacy. Due to the low overall quality of the original studies, more high-quality, large-sample, multi-center RCTs are needed to confirm its conclusions and provide a credible basis for clinical treatment.

## Author contributions

**Conceptualization:** Yongjing Wang, Liu xiuju.

**Data curation:** Yongjing Wang, Bokun Chen, Menghao Li, Mingxuan He, Yidan Huo.

**Formal analysis:** Yongjing Wang, Bokun Chen, Menghao Li, Xinhui Zhang.

**Writing – original draft:** Yongjing Wang, Bokun Chen, Menghao Li, Yunfei Tian.

**Writing – review & editing:** Yongjing Wang, Bokun Chen, Menghao Li, Yunfei Tian, Xinhui Zhang, Liu xiuju.

## References

[1] The Subspecialty Group of Infectious Diseases, the Society of Pediatrics, Chinese Medical Association. Expert consensus on the diagnosis and treatment of herpangina (2019). Chin J Pediatr. 2019;57:177–80. 10.3760/cma.j.issn.0578-1310.2019.03.004 30818893

[2] Linghui C Yuan D. Progress in traditional chinese medicine treatment of herpetic pharyngitis in children. China’s Naturpathy. 2021;29:120–3.

[3] Wenjuan L Zhang Q. Research progress on the mechanism and treatment of central nervous system lesionscaused by enterovirus EV71 infection. J Chengdu Med Coll. 2016;11:257–62.

[4] Yongji S. Progress in western medicine in pediatric herpetic pharyngitis. J Clin Med. 2017;4:18212–4.

[5] Yamei H Jiang Z. Futang Zhu Practice of Pediatrics. 7th ed. People’s Medical Publishing House; 2002.

[6] Xiaoming S Shen W. Pediatrics. 7th ed. People’s Medical Publishing House; 2008.

[7] Mudi W. Pediatrics. 5th ed. People’s Medical Publishing House; 2001.

[8] Xiaohong Z. Efficacy of ribavirin aerosol combined with Pudilan anti-inflammatory oral liquid in treating herpangina. Modern J Tradit Chin West Med. 2010;19:3742–3.

[9] Xiaoli W. Efficacy of ribavirin aerosol combined with Pudilan Anti-inflammatory Oral Liquid in treating herpangina. Contemp Med Symp. 2020;18:161–3.

[10] Fangli J Zhang G. Clinical effect of Pudilan anti-inflammatory oral liquid combined with interferon in the treatment of herpangina. Jilin Med J. 2015;36:3104–5.

[11] Meixing G. Observation on therapeutic effect of Pudilan anti-inflammatory oral liquid on herpangina. J Pract Tradit Chin Med. 2014;30:141.

[12] Zhanbiao L Ou M. Clinical effect of Pudilan anti-inflammatory oral liquid in the adjuvant treatment of herpangina. J Sichuan Tradit Chin Med. 2013;31:116–7.

[13] Ying Y. Pudilan anti-inflammatory oral liquid combined with ribavirin for the treatment of herpetic pharyngitis in 45 cases. J Shaanxi Univ Chin Med. 2018;41:100–1.

[14] Weiqin Y. Clinical analysis of Pudilan anti-inflammatory oral liquid in treating 40 cases of herpangina. Shenzhen J Integr Tradit Chin West Med. 2017;27:88–9.

[15] Haiqiang Z Lin A Ye E. The therapeutic effect of Pudilan anti-inflammatory oral liquid on 100 cases of herpangina in children. World Health Digest. 2013;10:188–9.

[16] Zhifang L. Clinical study on Pudilan Xiaoyan oral liquid in adjuvant treatment of children with herpetic angina. New Chin Med. 2023;55:156–60.

[17] Xiaoying Z Xie Q Chen F. Clinical observation on 36 cases of herpangina in children treated with Western medicine combined with XiaoerChiqiaoqingre Granule. Chin J Ethnomed Ethnopharm. 2018;27:103–104,116.

[18] Fangchun W Zhang F Wu H. Clinical study on treatment of herpangina in children with Xiaoer Chiqiaoqingre Granule granule combined with ribavirin. New Chin Med. 2021;53:94–7.

[19] Hua Z. Observation on the therapeutic effect of XiaoerChiqiaoqingre Granule on herpangina in children. Chin Pediatr Integr Tradit West Med. 2015;7:584–6.

[20] Suwen Y. Observation on the therapeutic effect of XiaoerChiqiaoqingre Granule on herpangina in children. World Latest Med Infor. 2017;17:115–16.

[21] Fengmei Z. Observation on the therapeutic effect of XiaoerChiqiaoqingre Granule on herpangina. Med Forum. 2016;20:2207–8.

[22] Xiaoli Z Tan X Ma J. Observation on the therapeutic effect of XiaoerChiqiaoqingre granule on herpangina. Chin Community Doctors. 2017;33:96–9.

[23] Xiaodong L. Study on therapeutic effect of XiaoerChiqiaoqingre Granule on herpangina. Chinese Journal of Modern Drug Application. 2019;13:108–9.

[24] Tianyang S Xiao M. Clinical study on treating herpangina with XiaoerChiqiaoqingre granule. New Chin Med. 2020;52:103–5.

[25] Wenshu X. Analysis of the clinical value of XiaoerChiqiaoqingre Granule in treating infantile herpangina. China Pract Med. 2021;16:134–7.

[26] Yu F. Clinical observation of XiaoerChiqiaoqingre Granule in the treatment of infantile herpangina. Jilin Med J. 2012;33:5008.

[27] Silu L. Observation on the therapeutic effect of XiaoerChiqiaoqingre Granule on herpangina in children. Chinese J Rural Med Pharm. 2019;26:22–3.

[28] Yanxia W Jin L. Clinical observation of XiaoerChiqiaoqingre Granulein treating herpangina. Zhejiang J Integr Tradit Chin West Med. 2012;22:119–20.

[29] Jing L. Clinical efficacy of interferon combined with XiaoerChiqiaoqingreGranule in the treatment of herpangina. J China Prescription Drug. 2019;17:128–9.

[30] Xiaoyu H Liu W. Efficacy of XiaoerChiqiaoqingreGranule in the treatment of herpangina. World Latest Med Infor. 2018;18:153–5.

[31] Runguo T. Clinical observation on herpangina treated with a combination of traditional Chinese medicine and Western medicine. Guangming J Chin Med. 2018;33:2109–10.

[32] Zhihua H Huang S. Effect of Xiaoer Chiqiao Qingre granules in adjuvant treatment of children with herpangina. Clin Med Eng. 2022;29:941–2.

[33] Cui Z Liu Q Yang J. Clinical efficacy of Xiaoer Chiqiao Qingre Granules combined with interferon in the treatment of herpangina and its impact on serum inflammatory factors. Heilongjiang Med J. 2023;36:105–7.

[34] Yingyi H Li X Li M. Influence of Xiaoer Chiqiao Qingre Granules Combined with Kangfuxin solution on children with herpangina. Chin Foreign Med Res. 2022;20:1–5.

[35] Jianyue S. Efficacy and safety of Lanqin Oral Liquid combined with ribavirin in the treatment of herpangina in children. Clin Educ General Pract. 2017;15:581–2.

[36] Dingbang S Ren W. Lanqin oral liquid combined with Vidarabine Monophosphate in the treatment of herpangina in children. Pract Clin J Integr Tradit Chin West Med. 2018;18:37–8.

[37] Zhiqiang H. Clinical observation of Lanqin Oral Liquid combined with ribavirin aerosol in the treatment of herpangina. Chin J Pharmacovigilance. 2011;8:711–2.

[38] Jianying L. Clinical observation on the treatment of herpangina by Lanqin Oral Liquid combined with routine Western medicine. China’s Naturopathy. 2020;28:95–7.

[39] Ling C Zhao W Sun Y. Effect of Lanqin Oral Liquid and Interferon α1b injection on inflammatory factors and humoral immunity in children with herpangina. Hainan Med J. 2021;32:3082–5.

[40] Ailin L Li L. Clinical observation of Lanqin Oral Liquid in the treatment of herpangina. Chin Pediatr Integr Tradit West Med. 2011;3:57–9.

[41] Wenyu W. Clinical efficacy and safety of Lanqin Oral Liquid in the treatment of herpangina in children. Chin J Modern Drug Application. 2018;12:113–4.

[42] Chenji X. Clinical effect and prognosis of Lanqin Oral Liquid on children with herpangina. Modern Pract Med. 2015;27:1199–201.

[43] Lijun H. The effect of Lanqin Oral Liquid as an adjuvant therapy for pediatric herpangina on clinical symptom improvement and inflammatory respons. Modern Diagn Treat. 2023;34:1608–10.

[44] Huanhuan W. Efficacy of Baicalin Oral solution combined with interferon alpha-2b in children with herpes pharyngitis and the effect on cellular immunity and inflammatory factors. J Rare Uncommon Dis. 2023;30:30–31 + 41.

[45] Xin L Zhang L. Analysis of the efficacy of lanqin oral liquid combined with inhalation of interferon-α in the treatment of children with herpetic angina. J Pract Tradit Chin Internal Med. 2023;37:133–6.

[46] Tingting Z. Effect of Lanqin oral solution combined with recombinant human interferon α2b spray in pediatric herpetic pharyngitis. Pract Clin J Integr Tradit Chin West Med. 2023;23:72–5.

[47] Suli M Wang Y. Effect of ribavirin spray combined with Shuanghuanglian Oral Liquid on herpangina in children. Chin Community Doctors. 2012;14:220.

[48] Jin X. Clinical study of Shuanghuanglian Oral Liquid combined with ribavirin nebulized inhalation in the treatment of herpangina in children. Clin J Chin Med. 2014;6:34–5.

[49] Youyun Y. Clinical analysis of Ribavirin spray combined with Shuanghuanglian Oral Liquid in the treatment of herpangina in children. Chin Community Doctors. 2012;14:43–4.

[50] Shuzhen M Kang J. Clinical observation on the treatment of 45 cases of herpangina in children with Lianhuaqingwen Granule combined with Western medicine. J Pediatr Tradit Chin Med. 2015;11:26–8.

[51] Lingyun Z. Clinical efficacy of Lianhuaqingwen Granule combined with antivirus in the treatment of herpangina in children. Chin J School Doctor. 2020;34:300–301, 311.

[52] Xiaojing L. Effect of Lianhuaqingwen Granule combined with Ribavirin on herpangina in children. Contemp Med Symp. 2021;19:164–5.

[53] Yanling W Zhou J Zhang Q. Effect of Lianhuaqingwen Granule on herpangina in children. Inner Mongolia J Tradit Chin Med. 2017;36:31–2.

[54] Chengxiang L. 37 cases of herpangina in children were treated with Shufengjiedu Capsule. Henan Tradit Chin Med. 2015;35:1695–7.

[55] Meilian Y. Clinical observation on 123 cases of herpangina in children treated with Shufengjiedu capsule. J Emerg Tradit Chin Med. 2016;25:2364–5.

[56] Yang Y Dongye L. The therapeutic effect of Shufengjiedu Capsule combined with recombinant interferon α1b on herpangina in children. J Emerg Tradit Chin Med. 2019;28:899–900.

[57] Haifan Z. Clinical observation on treatment of herpangina with XiaoerChaiguituire Granule combined with Western medicine. New Chin Med. 2016;48:188–9.

[58] Jiang W Cai K Wan M. The efficacy and safety of XiaoerChaiguituire granule in the treatment of herpetic angina and its effect on serum LGA and LGM. Hebei Med. 2019;25:1757–60.

[59] Yuhan L. Observation on curative effect of XiaoerNiuhuangqingxin powder on herpangina. Chin J Clin Rational Drug Use. 2013;6:82–3.

[60] Feng H Lu X Chen P. The curative effect observation of XiaoerShuangjinqingre Oral liquid on herpangina. Hubei J Tradit Chin Med. 2015;37:31.

[61] Zhichao X. Clinical observation of XiaoerShuangjinqingre Oral Liquid in the treatment of herpangina. China Med Pharm. 2014;4:90–91, 96.

[62] Yonghong D. Observation on the therapeutic effect of ribavirin granule combined with XiaoerShuangjinqingre oral liquid on herpangina. J Med Infor. 2014;27:397.

[63] Yingyu C. Clinical effect of XiaoerQingyan Granule combined with riboflavin sodium phosphate and interferon in the treatment of herpetic angina. Clin Res Pract. 2019;4:113–4.

[64] Dezhao Y. Clinical observation on 48 cases of herpangina treated with XiaoerQingyan Granule. New Chin Med. 2006;38:40–1.

[65] Xuanzi H. Clinical observation on treatment of herpangina in children with XiaoerQingyan Granule. J Henan Med Coll. 2022;34:53–5.

[66] Xin L Wang D. Effect of pediatric qingyan granule combined with Kaihoujian spray on children with herpetic angina. Clin Res. 2023;31:105–7.

[67] Suying G. Clinical observation on the treatment of herpangina in children with a combination of Chinese and Western medicine. J Pract Tradit Chin Med. 2020;36:1157–8.

[68] Zhiying L Zou X. Clinical observation on the treatment of 108 cases of herpangina in children with Kouyanqing Granule combined with Western medicine. J Pediatr Tradit Chin Med. 2019;15:45–7.

[69] Qinghua C He J Lu X. Antiviral effect of XiaoerShuangjinqingre oral liquid in vitro. J Pharmaceut Res. 2015;34:631–3.

[70] Xiaohui T Zheng Y Huang W. Progress in pharmacological and clinical studies of Pudilan preparation. J Yunnan Univ Tradit Chin Med. 2018;41:98–102.

[71] Lianxin W Miao Q Xie Y. Expert consensus statement on Pudilan Anti-inflammatory Oral Liquid in clinical practice. China J Chin Mater Med. 2019;44:5277–81. 10.19540/j.cnki.cjcmm.20191105.501 32237368

[72] Jimilihan SM. Network pharmacological study on the action mechanism of active compounds in Pudilan Anti-inflammatory Oral Liquid. J Shenyang Pharmaceut Univ. 2020;37:1117–24.

[73] Yujie Y Chang L. Advances in pharmacological research and clinical application of Lianhuaqingwen capsule/granule in respiratory diseases. Chin J Clin Pharmacol Ther. 2021;26:1174–80.

[74] Cheng L Liu H Wang T. Research progress on chemical constituents, pharmacological action, and clinical application of Lianhuaqingwen preparation. Chin Tradit Patent Med. 2021;43:3409–16.

[75] Xiaohua Y Gao F Zhang G. Bacteriostasis of Kouyanqing and its components in vitro. Chin J Microecol. 2015;27:260–2.

